# Fundamental roles of the Optineurin gene in the molecular pathology of Amyotrophic Lateral Sclerosis

**DOI:** 10.3389/fnins.2023.1319706

**Published:** 2023-12-21

**Authors:** Shumin Zhao, Ranran Chen, Ying Gao, Yanchao Lu, Xue Bai, Jingjing Zhang

**Affiliations:** ^1^Department of Neurology, Medical Research Center, Chifeng Municipal Hospital, Chifeng, China; ^2^Chifeng Clinical Medical College of Inner Mongolia Medical University, Chifeng, China

**Keywords:** optineurin, amyotrophic lateral sclerosis, mitophagy, neuroinflammation, protein aggregation

## Abstract

Amyotrophic lateral sclerosis (ALS) is a neurodegenerative disease characterized by the progressive loss of motor neurons (MNs) in the brain and spinal cord. It is caused by multiple factors, including mutations in any one of several specific genes. Optineurin (OPTN) mutation is an essential cause of some familial and sporadic ALS. Besides, as a multifunctional protein, OPTN is highly expressed and conserved in the central nervous system. OPTN exerts its functions by interacting with various proteins, often acting as an adaptor to provide a link between two or more core proteins related to autophagy and inflammation, etc. OPTN mutation mainly results in its function deficiency, which alters these interactions, leading to functional impairment in many processes. Meanwhile, OPTN immunopositive inclusions are also confirmed in the cases of ALS due to C9ORF72, FUS, TARDBP, and SOD1 mutations. Therefore, OPTN gene may play fundamental roles in the molecular pathology of ALS in addition to OPTN mutation. In this review, we summarize the recent advances in the ALS pathology of OPTN defect, such as mitophagy disorder, neuroinflammation, neuronal axonal degeneration, vesicular transport dysfunction, etc., which will provide a reference for research on the pathogenesis and treatment of ALS.

## 1 Introduction

Amyotrophic Lateral Sclerosis (ALS) is a fatal neurodegenerative disease characterized by progressive loss of upper and lower motor neurons (MNs). It has a prevalence of 1−3 cases per 1,00,000 people (Feldman et al., 2022). The average age of disease onset is 50−65 years old. Patients gradually develop progressive motor deficits for weeks or months, which can affect any voluntary muscle. Currently, there is no effective therapeutic agent for ALS except riluzole and edaravone, which could mildly delay the progression. Most patients die within 3−5 years due to respiratory failure. Clinically, familial ALS (fALS, 10% of cases) and sporadic ALS (sALS, 90% of cases) can be distinguished. More than 40 fALS mutated genes have been identified, including superoxide dismutase 1 (SOD1), Chromosome 9 Open Reading Frame 72 (C9ORF72), Transactive Response DNA Binding Protein 43 (TDP-43), Fused in Sarcoma (Fus), OPTN, and others (Kim et al., 2020). The pathogenesis of ALS is still unclear, which may include mitochondrial dysfunction, protein aggregation, abnormal neuroinflammation, vesicular transport disorders, axonal degeneration, and oxidative stress.

Mutations in the OPTN gene are associated with both fALS and sALS. Roughly 4% of fALS cases and 0.4% of sALS cases carry the OPTN gene mutation, which also causes several other genetic diseases including primary open-angle glaucoma (POAG), Crohn’s disease, and Paget’s disease (Minegishi et al., 2016). The frequency of the OPTN mutation carrier varies significantly among races, with higher rates observed in Asian populations, particularly in the Japanese population. This was demonstrated by Japanese researchers who identified the first case of an ALS patient associated with the OPTN mutation in Japanese individuals (Maruyama et al., 2010). OPTN mutations can cause ALS type 12 (ALS12), a slowly progressive, adult-onset disease that affects upper MNs and primarily affects the lower limbs. It can be inherited as an autosomal recessive or autosomal dominant manner (Mou et al., 2022). Regarding clinical manifestations, patients with the OPTN mutation tend to have limb involvement, which accounts for 75% of the patients. Medulla oblongata symptoms are observed in 20% of the patients, whereas cognitive deficits have relatively low incidence. OPTN-related ALS is characterized by a relatively slow progression and a long duration before respiratory dysfunction occurs, with an average age of onset of 45 years. It should be noted that familial and male patients often experience an earlier onset of the disease. Furthermore, in addition to the ALS phenotype, some patients with OPTN mutations may present with extrapyramidal symptoms, aphasia, or frontotemporal dementia (FTD). Table 1 provides a list of currently known OPTN mutation types and their associated clinical manifestations. This review aims to discuss the structure and function of OPTN and the possible mechanisms leading to the development of ALS.

**TABLE 1:** Optineurin mutation in the amyotrophic lateral sclerosis.

Nucleotide change	Protein change	Location	Variant type	Site of onset	Family history
c.7C→T	p.H3Y	Exon 4	Missense	Limbs	No
c.46C→G	p.P16A	Exon 4	Missense	Bulbar	No
c.67G→T	p.G23X	Exon 4	Non-sense	Lower limb	AD
c.177G→C	p.K59N	Exon 5	Missense	-	AD/AR
c.277G→C	p.A93P	CC Domain	Missense	Lower limb	No
c.287G→T	p.R96L	CC Domain	Missense	Lower limb	AD/AR
c.381_382insAG	p.D127Rfs*21	CC Domain	Missense	Lower limb, Cognition	AR
c.407C→T	p.A136V	CC Domain	Missense	Lower limb	No
c.481G→A	p.V161M	CC Domain	Missense	Upper limb	No
c.493C→T	p.Q165X	CC Domain	Non-sense	Bulbar, Upper limb	AD/AR
c.658delG	p.D220Mfs*12	Exon 8	Frameshift	Upper limb	AR
c.785C→A	p.S262X	CC Domain	Non-sense	Upper limb	AR
c.811C→T	p.R271C	CC Domain	Missense	Limbs	No
c.844A→C	p.T282P	CC Domain	Missense	Lower limb	No
c.883G→T	p.V295F	CC Domain	Missense	Upper limb	AD
c.941A→T	p.Q314L	CC Domain	Missense	Lower limb	No
c.964G→A	p.E322K	CC Domain	Missense	Bulbar	AD
c.1184A→G	p.K395R	CC Domain	Missense	Lower limb	No
c.1502C→T	p.Q398X	CC Domain	Non-sense	Bulbar, Upper limb	AR
c.1340T→G	p.M447R	CC Domain	Missense	-	No
c.1352T→C	p.I451T	CC Domain	Missense	Upper limb	No
c.1360C→G	p.Q454E	CC Domain	Missense	Bulbar	No
c.1289_1290del	p.L430Rfs*16	CC Domain	Frameshift	Upper limb	AR
c.1320delA	p.K440Nfs*8	CC Domain	Frameshift	Spinal	AD
-	p.M468R	UBD Domain	Missense	Bulbar, Cognition	AD
c.1433A→G	p.E478G	UBD Domain	Missense	Upper limb, Lower limb, Bulbar	AD
c.1442C→T	p.A481V	UBD Domain	Missense	-	AD/AR
c.1465A→G	p.K489E	UBD Domain	Missense	Bulbar, Upper limb	No
c.1481C→G	p.L494W	UBD Domain	Missense	Upper limb	No
c.1499T→C	p.L500P	UBD Domain	Missense	Aphasia	AD/AR
c.1546G→C	p.E516Q	Exon 4	Missense	Bulbar, Upper limb	No
c.1634G→A	p.R545Q	Exon 15	Missense	-	AD/AR
c.1670A→C	p.K557T	ZF Domain	Missense	Lower limb	AD
c.1690G→C	p.Q564H	ZF Domain	Missense	Lower limb	No
c.1704A→C	p.L568L	ZF Domain	Non-sense	Limbs	No
c.552 + 1delG	p.148_184del	Intron 6	Deletion	Lower limb	No
c.1401 + 4A→G	-	Intron 13	Deletion	Lower limb	No
-	Ex5del	Exon 5	Deletion	Upper limb	AR

AD, autosomal dominant; AR, autosomal recessive.

## 2 OPTN protein structure and function

**FIGURE 1:**
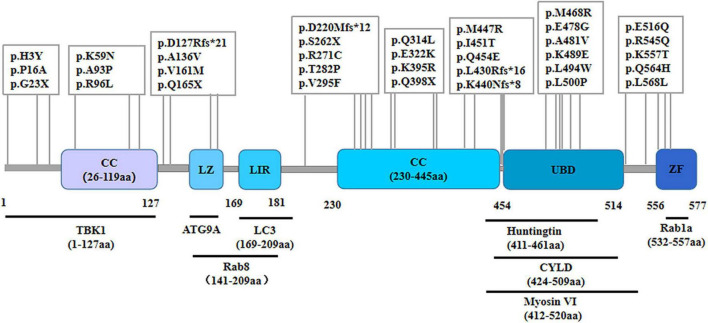
Schematic representation of the OPTN protein and specific mutant sites. The OPTN protein is a scaffold protein of 577 amino acids containing several structural domains, including two coiled-coil (CC) domains, a leucine zipper (LZ) domain, an LC3 interaction region (LIR) domain, a ubiquitin-binding (UBD) domain, and a zinc finger (ZF) domain. It shows various points of mutation and the positions within the molecule where it binds to multiple interacting proteins.

As a cytosolic protein, OPTN protein is genetically highly conserved and has identical sequences in various species, including humans, mice, rats, and pigs. The human OPTN protein is encoded by a locus on chromosome 10p13. It is a 74-kilodalton scaffolding protein comprising 577 amino acids and is expressed in several tissues, such as the brain, liver, and heart (Qiu et al., 2022). OPTN proteins contain multiple structural domains, including CC, LZ, LIR, UBD, and ZF (Figure 1). The CC structural domain can selectively recognize protein aggregation and bind to them independently of ubiquitination, contributing to the process of mitophagy. The LIR structural domain serves as the binding site of LC3-II, enabling OPTN to connect ubiquitination-tagged targets with the autophagy-critical protein LC3, facilitating the formation of autophagosomes and participating in selective autophagy, knockout of OPTN leads to decreased autophagic activity in mice (Fukushi et al., 2023). As an autophagy adapter, UBD selectively binds to degraded substances such as damaged mitochondria, protein aggregations, or intracellular pathogens, and transports them to autophagosomes. The LZ structural domain interacts specifically with ATG9A, promoting the degradation of ubiquitin-labeled mitochondria. OPTN, a homolog of NF-κB essential regulator (NEMO), competitively inhibits the NF-κB pathway and TNF-α production through its UBD domain (Slowicka and van Loo, 2018). Additionally, OPTN can interact with Myosin VI, Rab8, Huntingtin, and TANK-binding protein 1 (TBK1) through its UBD, LZ and LIR domains, CC domain, and NEMO-like domain, respectively. These interactions contribute to its role as a multifunctional protein involved in various cellular processes, including vesicular trafficking, inflammatory and antiviral signaling, anti-bacterial responses, and autophagy. Disease-causing mutations in OPTN may disrupt these interactions, leading to abnormal signaling.

## 3 The role of OPTN in ALS pathology

### 3.1 OPTN defect results in mitophagy disorder

Mitophagy, a form of selective autophagy, is essential for preserving intracellular homeostasis as it selectively eliminates dysfunctional or surplus mitochondria, protecting cells against mitochondrial stress. Among the ubiquitin-dependent pathways, the most widely studied is the PTEN-induced putative kinase protein-1 (PINK1)/Parkin pathway. When mitochondria are damaged, the mitochondrial membrane potential decreases, preventing PINK1 from entering the inner mitochondrial membrane. As a result, PINK1 accumulates in the outer mitochondrial membrane, leading to the recruitment and activation of Parkin. Activated Parkin undergoes a spatial conformation change and converts into an activated E3 ubiquitin ligase. This ligase then ubiquitinates several outer membrane components. Subsequently, autophagy receptor proteins, such as p62, OPTN, and NDP52, accumulate in the mitochondrial outer membrane. These proteins facilitate the recruitment of ubiquitination products into autophagosomes by binding to LC3. Mature autophagosomes then fuse with lysosomes to form autophagic lysosomes, leading to the degradation of the contained mitochondria. In addition to the ubiquitination pathway, there is another pathway that initiates mitochondrial autophagy. In this non-ubiquitination pathway, proteins like Nip3-like protein X (NIX), bcl2-interacting protein 3 (BNIP3), and FUN14 domain containing 1 (FUNDC1) are recruited to autophagosomes. These proteins can directly bind to LC3 without ubiquitination, thereby initiating mitochondrial autophagy.

The accumulation of impaired or malfunctioning mitochondria is a significant contributing factor to the pathogenesis of ALS. Essential contributors to mitochondrial dysfunction in ALS include mutated genes such as SOD1 and TBK1, as well as other genetic factors. In spinal cord, autopsy samples of individuals with ALS, neurons containing the TDP-43 protein inclusion body exhibited a decreased presence of Parkin, a crucial protein involved in mitophagy (Lagier-Tourenne et al., 2012). Furthermore, TBK1 mutation or deletion impeded the recruitment of OPTN and LC3 to impaired mitochondria, thereby preventing the initiation of mitophagy and resulting in the accumulation of damaged mitochondria. Consequently, this accumulation of damaged mitochondria is responsible for the development of ALS disease (Gerbino et al., 2020). Research has shown that the PINK1-Parkin pathway is necessary for mitophagy to reduce damage in distant axons. By bypassing the need for transportation to somatic cells, localized mitophagy can offer quick neuroprotection against oxidative stress (Ashrafi et al., 2014). Therefore, it is clear that mitophagy is a vital factor in the development of ALS and is exclusive to the neuronal cytosol.

Optineurin (OPTN) is an autophagy receptor that selectively binds intracellular bacteria, damaged mitochondria, and aggregates of mutant proteins (Weil et al., 2018; Padman et al., 2019). Parkin ubiquitination recruits OPTN to damaged mitochondria, prompting its binding. Subsequently, LC3 is recruited to create autophagic vesicles encircling the affected mitochondria. Mutations in OPTN decrease the efficiency of mitophagy, leading to the accumulation of damaged mitochondria (Shen et al., 2015). MNs mortality occurs due to reduced autophagy phagocytosis and degradation of damaged mitochondria caused by the unstable binding of OPTN-related UBAN, E478G, and Q398X mutants to these mitochondria (Wong and Holzbaur, 2014; Moore and Holzbaur, 2016). SOD1 mutant aggregates can recruit and segregate a significant number of OPTNs, resulting in a decrease in mitochondrial autophagic flux. OPTN dysfunction also aggravated mitophagy dysfunction caused by SOD1 mutations. Exogenous enhancement of OPTN expression can mitigate the toxicity of the mutant SOD1 protein in the neuron (Tak et al., 2020). In conclusion, ALS may be caused by OPTN mutations or loss of function through the mitophagy pathway.

### 3.2 OPTN contributes to protein aggregation in ALS

A significant feature of ALS is the presence of ubiquitinated inclusion bodies in neurons and glial cells, which are pathological protein aggregations. Most ALS patients have neuronal cytoplasmic ubiquitinated inclusion bodies, as confirmed by histological analysis of spinal cord samples. Pathological protein aggregations were seen in all mutations in the SOD1, VCP, and UBQLN2 proteins, with TDP-43 being the predominant component of ubiquitinated protein aggregations in sALS patients (Neumann et al., 2006). The cerebrospinal fluid of patients with sALS contains misfolded SOD1 aggregates that can cause apoptosis in motor neuron-like NSC-34 cells (Tokuda et al., 2019). Aggregation of mutant SOD1 protein can lead to neuronal cell death by sequestering vital survival proteins. Reducing protein aggregation in neurons and glial cells through various mechanisms, including enhancing the autophagic lysosomal pathway, is vital in the treatment of ALS (Zhang et al., 2014; Zhang et al., 2018).

In ALS patients, specific OPTN protein aggregations form in the cytoplasm and axons of MNs located in the anterior horn of the spinal cord, thereby disrupting protein homeostasis (Maruyama et al., 2010). Some fALS and sALS patients show the presence of OPTN co-localized with TDP-43, FUS, and SOD1 in neuronal inclusion bodies. OPTN mutations can enhance ubiquitinated TDP-43 expression in neuronal cytoplasm. This occurs through increased T-cell cytoplasmic antigen 1 expression, which induces the aggregation of ubiquitinated TDP-43 (Kakihana et al., 2021). OPTN deletion enhances TDP-43 protein expression in microglia, while OPTN overexpression reduces aggregation of SOD1 mutant protein (Tak et al., 2020). OPTN activates the autophagy-lysosome pathway and eliminates protein aggregations that are not attached to ubiquitin, using its CC structural domain. If the CC structural domain of OPTN is damaged, it could not interact with mutant protein aggregations anymore. Therefore, OPTN might contribute to the elimination of protein aggregation formed during the development of ALS.

### 3.3 OPTN involved in neuroinflammation in ALS

Neuroinflammation plays a significant role in the progression of ALS. TLR4 signalling pathway genes have been shown to be up-regulated in peripheral blood mononuclear cells from early-stage ALS patients. Furthermore, microglia, dendritic cells, and lymphocytes are observed to be activated and infiltrate the spinal cord and cerebrospinal fluid in late-stage ALS patients (Henkel et al., 2004; Philips and Robberecht, 2011). Mutations in ALS-related genes like SOD1, TBK1, C9ORF72, and PGRN can induce neuroinflammatory responses. The mutant SOD1 protein can activate inflammatory vesicles in microglia, leading to the initiation and worsening of the neuroinflammatory response, with the extent of activation dependent on dosage and time (Deora et al., 2020). Additionally, the TBK1 mutation impacts type I interferon production and T-cell migration, thereby exacerbating the neuroinflammatory damage seen in ALS. In the context of ALS, neuroinflammation plays a dual role. Initially, anti-inflammatory cytokines provide neuroprotection in the early stages of the disease. However, as the disease progresses, microglia and astrocytes become activated, releasing proinflammatory cytokines. This activation creates a neurotoxic environment that ultimately leads to MNs death. Hence, neuroinflammation in ALS can be perceived as a double-edged sword (Beers and Appel, 2019). Modulating the neuroinflammatory response could benefit ALS treatment.

Mutations in the OPTN gene affect the neuroinflammatory response through loss of function. Additionally, OPTN plays a crucial role in inhibiting NF-κB activity, which is essential for the innate immune response. When OPTN is absent or mutated, NF-κB moves to the nucleus and increases the expression of numerous proinflammatory genes, leading to the subsequent amplification of microglia-mediated neuroinflammation. The NF-κB immunoreactivity is reduced in neurons and elevated in microglia in the spinal cords of fALS and sALS patients with OPTN mutations (McCauley and Baloh, 2019). In addition, excessive production of IFNβ has been observed in cells and mice with knocked-down OPTN, as well as in patients with OPTN mutations following viral infection (Fukushi et al., 2023). Mice infected with an OPTN mutant lentivirus displayed ALS-like symptoms marked by cortical inflammation activation and increased secretion of proinflammatory cytokines (Liu et al., 2018). ALS mouse models lacking the OPTN UB binding region showed reduced TBK1 activation and interferon production when stimulated with lipopolysaccharide, which triggered microglial activation and caused neuronal apoptosis (Markovinovic et al., 2018). Thus, OPTN mutation or loss of function may significantly contribute to the development of ALS.

### 3.4 OPTN defect disrupts vesicular transport in ALS

Vesicular transport is a fundamental physiological process involving the use of vesicles by cells to transport substances either between the cell and its environment or between different organelles within the cell. Impaired vesicular transport is crucial in the development of neurodegenerative diseases. As a neurodegenerative disease, ALS is associated with numerous pathogenic genes involved in vesicular transport, including SOD1, FUS, C9ORF72, and ANXA11. Impaired vesicular transport has been observed in the MNs of ALS mice. Mutant SOD1G93A mice, which exhibit reduced glutamate uptake, induce impaired vesicular transport due to auto excitotoxicity. This excitotoxicity leads to elevated mRNA and protein levels of vesicular glutamate transporter protein 2 at the synaptic terminals of perisynaptic cortical neurons (Saba et al., 2016). It was proposed that hexanucleotide repeat amplification of the C9ORF72 gene caused severe problems with intracellular and extracellular vesicular transport in fibroblasts and MNs. However, reducing the problems with vesicular transport by using vesicularly transported PLKfyve kinase inhibitors slows down MNs degeneration caused by different types of ALS mutations (Shi et al., 2018; Hung et al., 2023).

Myosin VI is a vital transport protein found in cytosolic, secretory vesicles, and autophagosomes. Additionally, OPTN is an articulating protein that co-localizes with Myosin VI at the Golgi and plasma membranes. OPTN knockdown results in the loss of Myosin VI from the Golgi complex, division of the Golgi complex, and impaired vesicular transport. The interaction between Rab8 and HTT proteins plays an important role in the recruitment of OPTN proteins associated with vesicular transport. OPTN proteins act as binding chaperones for Rab8 and HTT proteins. The activated form of Rab8 interacts with the amino-terminal region of OPTN, while HTT proteins bind to the carboxy-terminal end of OPTN. This binding of OPTN and HTT proteins enhances the recruitment of HTT to Rab8-positive vesicular structures, thereby regulating the vesicular transport process (Hattula and Peränen, 2000). There is a significant decrease in the proportion of OPTN-colocalized vesicles and decreased binding of OPTN to Myosin VI in MNs of autopsy spinal cord tissue from sALS patients. Furthermore, OPTN mutations that hinder its binding to Myosin VI result in abnormal cytoplasmic distribution, disrupted secretory protein transport, endoplasmic reticulum stress, and Golgi apparatus fragmentation in motor neuron-like NSC-34 cells (Sundaramoorthy et al., 2015). OPTN potentially contributes to ALS by impairing vesicular transport.

### 3.5 OPTN functional loss induces neuronal axonal degeneration

Neuronal axonal degeneration is an essential mechanism in the pathogenesis of ALS. Studies have found that manifestations of axonal degeneration exist before the apoptosis of ALS MNs. ALS-causing genes, such as SOD1, TDP-43, FUS, and DCTN, regulate neuronal axonal degeneration and regeneration. During the progression of ALS, Deng et al. (2018) showed that SOD1 mutations leaded to axonal and myelin degeneration, ultimately causing decreased locomotor performance in mutant mice at the advanced disease stage. Furthermore, the formation and regeneration of axonal myelin were impaired in mutant SOD1. Mutations in TDP-43 cause the gradual degeneration of adult Drosophila’s MNs and neuromuscular junctions. This degeneration is accompanied by the emergence of transverse striations within axons and the gradual loss of structural integrity (Sreedharan et al., 2015). Knocking down SARM1 in a TDP-43 mutant ALS mouse model reduces MNs axonal degeneration, denervation of neuromuscular junctions, and enhances the survival of ALS mice (White et al., 2019). Therapies that delay axonal degeneration and function loss protect against ALS progression.

Deleting the OPTN gene contributes to progressive myelin abnormalities and axonal degeneration through its involvement in the necroptosis mechanism of the central nervous system, ultimately resulting in ALS. RIPK1, RIPK3, and MLKL play significant roles in apoptosis and necroptosis, acting as crucial regulators. These molecules were identified in both SOD1G93A transgenic mice and human ALS pathology samples. The study demonstrates that loss of OPTN function leads to the activation of RIPK1-dependent signaling, resulting in progressive myelin and axonal degeneration in the CNS through a necrotic mechanism (Ito et al., 2016). Therefore, restoration of OPTN gene function may prevent the early axonal degeneration of MNs in ALS.

### 3.6 OPTN mutations increase oxidative stress

Oxidative stress is an initial concern in the development of ALS and may work alongside other mechanisms, such as protein aggregation and mitochondrial dysfunction, to exacerbate MNs injury. Patients with early ALS have elevated levels of markers of oxidative stress damage in various body fluids including plasma, urine, and cerebrospinal fluid. These markers, such as 8-hydroxy-29-deoxyguanosine (8OH29dG), protein carbonyls, and Reactive Oxygen Species (ROS), are also found in the spinal cord and motor cortex of autopsy samples taken from individuals in late-stage ALS (Gerovska et al., 2023; Olufunmilayo et al., 2023). Mutations in ALS genes, such as SOD1, TDP-43, APEX1, HFE, and PON, lead to oxidative stress damage and worsen mitochondrial degeneration. The SOD1G93A mutation in cellular models activates the MPO/HOCl pathway, leading to iron enrichment, lipid peroxidation, and irreversible apoptosis (Peng et al., 2022). Mutant TDP-43 cytoplasmic aggregation in neurons augments neuronal injury, hastening ALS progression through heightened intracellular oxidative stress. Riluzole and edaravone are therapeutic agents that exert neuroprotective effects by inhibiting oxidative stress injury in ALS.

Optineurin (OPTN) primarily regulates mitochondrial quality control, which is closely associated with oxidative stress. SH-SY5Y cells carrying an OPTN mutation displayed reduced activity of antioxidant enzymes and increased levels of reactive oxygen species (ROS), leading to impaired mitochondrial function and neuronal death. The overexpression of wild-type OPTN attenuated oxidative stress-related injuries in neurons caused by hydrogen peroxide (Zhu et al., 2016). According to studies, the production of reactive oxygen species by oxidative stress stimulates the production of mitophagy, which protects cell viability by mitigating the damage caused by oxidative stress and eliminating damaged mitochondria. However, if the level of oxidative stress surpasses the threshold that mitophagy can tolerate, damaged mitochondria accumulate. Within hours, OPTN can mediate the mitophagy pathway, effectively isolating damaged mitochondria and maintaining neuronal homeostasis. Nevertheless, studies have shown that OPTN mutations induce the production of reactive oxygen species, resulting in long-term oxidative stress injury and massive mitochondrial damage. Under these circumstances, mitophagy is unable to compensate for the removal of damaged mitochondria, leading to the formation of protein aggregations and the subsequent development of ALS disease. Consequently, it is suggested that OPTN mutations may contribute to the pathogenesis of ALS through oxidative stress injury.

## 4 Foresight

**FIGURE 2:**
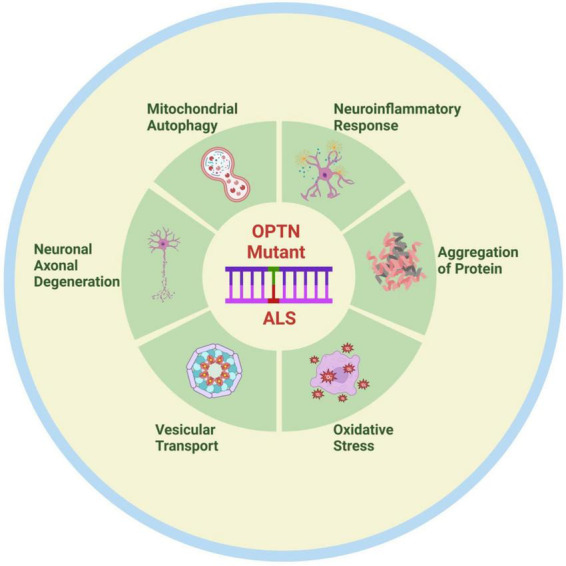
Possible pathogenesis of OPTN mutation-associated ALS. OPTN mutations contribute to the onset and progression of ALS through various mechanisms, including mitophagy disorder, neuroinflammation, protein aggregation, vesicular transport disorders, axonal degeneration, and oxidative stress. Created by BioRender.com.

As the functions of OPTN have been studied in more detail, new functions of OPTN have gradually been discovered. For instance, OPTN dysfunction regulates bone lipid homeostasis in the bone marrow cavity via the substrate fatty acid binding protein 3 (FABP3), leading to the onset of senile osteoporosis, and OPTN mutations maybe result in lipid metabolism abnormalities in patients and models of ALS. Unfortunately, there is currently little research into the treatment of ALS due to OPTN deficiency. One study found that neutralizing peripheral circulating IL1β could regulate neuroinflammation and improve motor function in OPTNE478G mouse model (Hu et al., 2023). Another study also found that blocking RIPK1 may provide a treatment option for OPTN mutation-mediated ALS by inhibiting axonal degeneration caused by inflammation and necroptosis (Ito et al., 2016). In brief, OPTN mutations primarily cause ALS by under expressing and causing complete loss of function of the expression product, which could impact ALS onset and progression via pathways including mitophagy, protein aggregation, neuroinflammation, vesicular transport, axonal degeneration, and oxidative stress (Figure 2). This observation may provide important insights into the pathogenesis of ALS. A thorough investigation is also needed to determine whether the OPTN protein is implicated in the pathogenic mechanisms of other ALS gene mutations. With the recent discovery of OPTN cell function and the gene mutation mechanism of ALS, new ideas and directions are anticipated to emerge for ALS pathogenesis and therapeutic research.

## Author contributions

The idea for this review topic was conceived by JZ and XB, who also contributed to revising the manuscript. SZ and RC were responsible for designing and drafting the manuscript and figures. YG and YL took part in the article writing discussion and were involved in drafting and modifying the table. All authors made contributions to revising the manuscript and approved the submitted version.
